# Application of YOLOv4 for Detection and Motion Monitoring of Red Foxes

**DOI:** 10.3390/ani11061723

**Published:** 2021-06-09

**Authors:** Anne K. Schütz, Verena Schöler, E. Tobias Krause, Mareike Fischer, Thomas Müller, Conrad M. Freuling, Franz J. Conraths, Mario Stanke, Timo Homeier-Bachmann, Hartmut H. K. Lentz

**Affiliations:** 1 Friedrich-Loeffler-Institut (FLI), Federal Research Institute for Animal Health, Institute of Epidemiology, Südufer 10, 17493 Greifswald-Insel Riems, Germany; Anne.Schuetz@fli.de (A.K.S.); Franz.Conraths@fli.de (F.J.C.); Timo.Homeier@fli.de (T.H.-B.); 2 Friedrich-Loeffler-Institut (FLI), Federal Research Institute for Animal Health, Institute of Animal Welfare and Animal Husbandry, Dörnbergstr. 25/27, 29223 Celle, Germany; verena_schoeler@yahoo.de (V.S.); Tobias.Krause@fli.de (E.T.K.); 3 Institute of Mathematics and Computer Science, University of Greifswald, Walther-Rathenau-Straße 47, 17487 Greifswald, Germany; mareike.fischer@uni-greifswald.de (M.F.); mario.stanke@uni-greifswald.de (M.S.); 4 Friedrich-Loeffler-Institut (FLI), Federal Research Institute for Animal Health, Institute of Molecular Virology and Cell Biology, Südufer 10, 17493 Greifswald-Insel Riems, Germany; Thomas.Mueller@fli.de; 5 Friedrich-Loeffler-Institut (FLI), Federal Research Institute for Animal Health, Südufer 10, 17493 Greifswald-Insel Riems, Germany; Conrad.Freuling@fli.de

**Keywords:** YOLOv4, red foxes, animal activity, computer vision, animal monitoring

## Abstract

**Simple Summary:**

The use of surveillance videos of animals is an important method for monitoring them, as animals often behave differently in the presence of humans. Moreover, the presence of humans can be a source of stress for the animals and can lead to changes in behavior. Extensive video material of red foxes has been recorded as part of a vaccine study. Since manual analysis of videos is both time-consuming and costly, we performed an analysis using a computer vision application in the present study. This made it possible to automatically analyze the videos and monitor animal activity and residency patterns without human interference. In this study, we used the computer vision architecture ‘you only look once’ version 4 (YOLOv4) to detect foxes and monitor their movement and, thus, their activity. Computer vision thereby outperforms manual and sensor-based exhaustive monitoring of the animals.

**Abstract:**

Animal activity is an indicator for its welfare and manual observation is time and cost intensive. To this end, automatic detection and monitoring of live captive animals is of major importance for assessing animal activity, and, thereby, allowing for early recognition of changes indicative for diseases and animal welfare issues. We demonstrate that machine learning methods can provide a gap-less monitoring of red foxes in an experimental lab-setting, including a classification into activity patterns. Therefore, bounding boxes are used to measure fox movements, and, thus, the activity level of the animals. We use computer vision, being a non-invasive method for the automatic monitoring of foxes. More specifically, we train the existing algorithm ‘you only look once’ version 4 (YOLOv4) to detect foxes, and the trained classifier is applied to video data of an experiment involving foxes. As we show, computer evaluation outperforms other evaluation methods. Application of automatic detection of foxes can be used for detecting different movement patterns. These, in turn, can be used for animal behavioral analysis and, thus, animal welfare monitoring. Once established for a specific animal species, such systems could be used for animal monitoring in real-time under experimental conditions, or other areas of animal husbandry.

## 1. Introduction

Animal welfare plays an increasingly important role in areas of animal husbandry and animal experimentation. Monitoring animal activity is one way to draw conclusions about welfare [1]. A change in activity may be triggered by a variety of factors including disease [2] or animal welfare issues. In particular, changes of behavioral activity can give information about the welfare or a disease situation of an animal [3,4]. Observing, measuring, and evaluating animal behavior are important indicators to determine the welfare status of animals [5]. The fact that animals often behave differently in the presence of humans may cause bias [4,6,7,8]. Moreover, humans are often not available all day for observations, so that the time is limited, during which animals can be observed without gaps. Therefore, monitoring methods that allow observing, measuring, and analyzing the activity behavior of animals in absence of humans are needed.

Furthermore, an automated monitoring system can be useful for continuous monitoring to detect specific or irregular events [9]. Radio-frequency identification (RFID) technology can be used for automated animal monitoring [10,11] or specific space use [12]. To this end, an RFID tag is implanted or embedded in an ear tag, collar, or leg band [13]. Another method is the use of accelerometers. Dutta et al. [2] used collar sensors with 3-axis accelerometer and magnetometer for cattle. Robert et al. [14] utilized accelerometer data from leg sensors of cattle to classify activities like lying, standing, or walking. Yet, RFID systems and sensors, like accelerometers, require certain interventions, like the implantation of a RFID chip or equipping the animal with a RFID tag or a sensor. These interventions and wearing these devices may cause stress for the animals [15] and could have an effect on their behavior.

For these reasons, the analysis of unmanipulated animals using video material or images represents an effective tool to obtain information about health- or welfare-related indicators, such as activity pattern changes [16]. However, visual inspection of videos is time consuming, measuring may be difficult, and the interpretation of the observations is prone to bias [3]. Hence, a combination of digital video and computer vision techniques is a non-stressful, non-invasive, cost effective, and easy method for monitoring animal behavior [15] that allows largely unbiased measurements and analyses of animal activities. Object detection methods classify objects and determine their position in images or videos. In the field of computer vision, many efficient algorithms have already been developed for the detection of objects, such as the human face [17]. Deep learning models, and, in particular, the use of convolutional neural networks (CNN), becomes increasingly important. Carl et al. [18] used a pre-trained FasterRCNN+InceptionResNetV2 network for automated detection of European wild mammal species. Ratnayake et al. [19] applied background subtraction and together with deep learning-based detection to detect and track honeybees.

Fernández-Carrión et al. [20] used a CNN to detect a collar of a wild boar to determine its daily motion. This daily motion can be used to differentiate between sick and healthy animals and may, thus, help to facilitate early detection of a disease.

A comprehensive animal detection without sensor equipment, in combination with activity determination is missing so far. For complete monitoring, different camera recordings need to be evaluated simultaneously.

Here, we demonstrate an application of deep learning for the detection and tracking of red foxes (*Vulpes vulpes*) during an experimental study. The latter was originally conducted to measure the long-time immunogenecity of a vaccine in red foxes being a reservoir for rabies [21]. We focus on the video surveillance data being a byproduct of this study.

In order to monitor the behavior of the animals automatically, we trained a CNN (YOLOv4) for fox detection. You only look once (YOLO) is a one-stage object detection algorithm for real-time object detection using convolutional neural networks (CNN) [22,23]. YOLOv4 consists of a ‘backbone’, a ‘neck’ and a ‘head’ [24]. The backbone is a CSPDarknet53, an open source neural network framework, to train and extract features [23,24]. The neck is a path aggregation network (PAN) and spatial pyramid pooling (SPP) used to collect feature maps from different stages [24]. The head, YOLOv3 [23], is used to implement object detection [24]. Bochkovskiy et al. [24] showed that YOLOv4 is a state-of-the-art detector, which is faster and more accurate than other available detectors.

The results of this detection can be used to infer different movement patterns, which can then be used to distinguish between different activity levels. The presented technique can be applied to detect movement patterns of animals, including active and inactive behavior. The generated daily, weekly, or monthly activity overviews can be used to monitor the animal activity, and may, thus, represent potential indicators for animal welfare.

## 2. Materials and Methods

### 2.1. Experimental Setup

**Figure 1:**
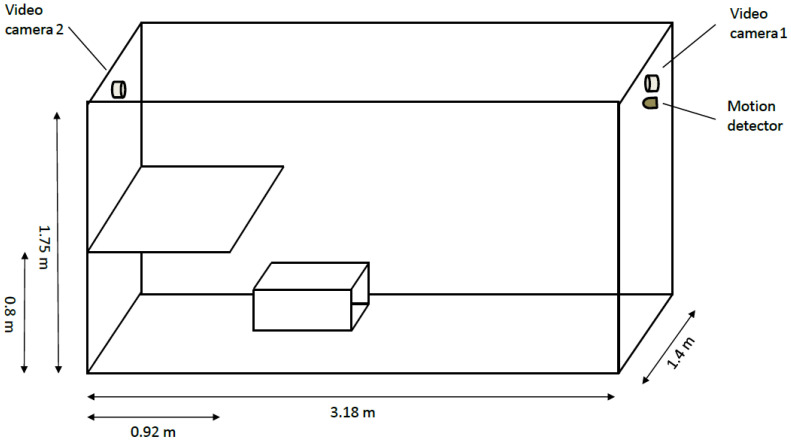
Illustration of the dimensions of the cage in which foxes were housed.

The data used in this paper are a subset of data generated in an experimental study with foxes conducted over 450 days [21]. A total of 23 red foxes (*Vulpes vulpes*) of the fur color variant ‘silver fox’ [25] were separately kept in cages, sized 318 cm × 140 cm × 175 cm (length × width × height), equipped with a platform, 92 cm × 140 cm (length × width) at height of 80 cm above the bottom of the cage (Figure 1).

Animal housing and maintenance were in accordance with national and European legislation and followed the guidelines for the veterinary care of laboratory animals [26]. The study was approved by the local authority in Mecklenburg-Western Pomerania (Landesamt für Landwirtschaft, Lebensmittelsicherheit und Fischerei Mecklenburg-Vorpommern, # FLI-7221.3-1-087/16). One of the requirements for approval was the availability of external monitoring including recording using video cameras. To this end, all animals were monitored by two cameras (ABUS IR HD TVIP61500, ABUS, Wetter, Germany). Additionally one infra-red motion detector (LBM 926, GEV GmbH, Ahrensburg, Germany), which was connected to a data logger (EL-USB-3, Lascar Electronics, Wiltshire, UK) and registered motion in 10 s intervals in each cage, was installed. The experimental study was conducted at the Friedrich-Loeffler-Institut (FLI), Greifswald-Insel Riems, Germany. Overall, 33 TB of video material was recorded discontinuously, for a total of 73 days.

### 2.2. Manual Evaluation and Motion Detector Data

Initially, video material was evaluated only manually. To this end, one person watched a short time period of 30 s before and after the sample point (of one or both cameras) every 15 min and entered the observed behavioral activity into a table. From this manually extracted data, the classification into ‘activity’, ‘no activity’, or ‘blind spot’ could be extracted.

Additionally, for the motion detector data, every 10 s it was recorded whether the motion detector detected a movement within the last 10 s or not. The motion detection data were recorded live during the experiments.

### 2.3. Image and Video Data

The video material has a resolution of 1280 pixels (horizontal) × 720 pixels (vertical) and of 15 frames per second (fps). An image set was extracted from the video material. The image set consists of images of different foxes (all 23) and with different body postures. Furthermore, images with different illumination conditions, like day and night, were selected. The complete image set consists of 7363 images, 4467 day scenes and 2896 night scenes. The software LabelImg [27] was used to manually label the foxes in each image of the image set. The image set was split into a training (80%—5890 frames) and a test set (20%—1473 frames), maintaining the relation of day and night scenes. The training set was used to train a YOLOv4 object detection algorithm and the test set to evaluate the trained fox detection.

### 2.4. Environment Configuration

Graphic card: NVIDIA K80 with 2 GPUs and 24 GB video RAM (nvidia, Santa Clara, CA, USA)

Operating system: CentOS 8

Processor: Intel Xeon E5-2667 v4 with 3.20 GHz

RAM: 377 GB

The algorithm was developed by using a jupyter notebook [28] and Python 3.6.8 [29].

### 2.5. Automatic Evaluation of Video Data: Fox Detection

The detection of red foxes is performed by using the deep learning algorithm YOLOv4. Pre-trained YOLOv4 weights for general-purpose object detection on photos were used to initialize transfer learning using the training set for fox detection. In this paper, we restrict the possible detected classes to foxes exclusively, and, therefore, train the network using a single class output only.

The training was implemented following the instructions of the YOLOv4 Github page [30] with pre-trained weights (that is, a pre-trained weights-file yolov4.conv.137, downloaded from GitHub [30]) and the parameters shown in Table 1.

In this part we give a detailed description on how to train the algorithm. Experienced users of the software can skip this part and continue after step 8. The algorithm was trained as follows:

1. Download and extract YOLOv4 from GitHub [30];
2. Copy the content of cfg/yolov4-custom.cfg to the new created file yolo-obj.cfg and change the following lines:
  line 3:  batch=64 
  line 4:  subdivisions=1 
  line 8:  width=416 
  line 9:  height=416 
  line 20:  max_batches=2000  (classes×2000)
  line 22:  steps=1600,1800  (80 and 90% of maxbatches)
  lines 603, 689, 776:  filters=18 ((classes+5)×3)
  lines 610, 696, 783:  classes=1 
3. Create a file obj.names with the name of each object in separate lines, here the file has only one line:
  fox
4. Label each image of the image set, such that for each image there exists a .txt file with the following values for every labeled object:
  <object-class> <BB x_center> <BB y_center> <BB width> <BB hight>
  with <object-class> an integer between 0 and number of classes − 1, and <BB x_center>, <BB y_center>, <BB width>, and <BB hight> are float values between (0,1], relative to the image height and width. Thus, the directory with the images contains a .txt file for each image with the same name.
  Create the files train.txt and test.txt. Split the image set into a training and test set and save the file names of the images, with respect to the full path relative to the directory darknet, in the respective file (one file name per line).
5. Create a file obj.data containing the number of classes and paths to train.txt, obj.names, and the backup folder:
  classes = 1
  train = data/train.txt
  names = data/obj.names
  backup = backup/
6. For starting the training run the code:
  ./darknet detector train obj.data yolo-obj.cfg yolov4.conv.137
  The training can take several hours. During training the trained weights are saved in the backup/ directory, yolo-obj_xxxx.weights every 1000 iterations and yolo-obj_last.weights every 100 iterations. After training the final weight, yolo-obj_final.weights is also stored there.  
7. Evaluate the results for trained weights:
  ./darknet detector map obj.data yolo-obj.cfg backup/yolo-obj_final.weights
8. Using the trained detector:
  ./darknet detector test obj.data yolo-obj.cfg backup/yolo-obj_final.weights

For the evaluation of the model performance the following values were determined: mean average precision (mAP), precision, recall (Equations (1)–(3), respectively), and detection speed.

(1) $$mAP=\frac{\sum_{c=1}^{C}AP(c)}{C}$$

(2) $$precision=\frac{TP}{TP+FP}$$

(3) $$recall=\frac{TP}{TP+FN}$$

with AP: average precision, *C*: number of classes, TP: number of true positive, FP: number of false positive, FN: number of false negative. The AP is determined using the interpolated average precision as described in Everingham et al. [31]: First, the detected BBs of a class are ranked according to their confidence and determined whether being true positive (TP) or false positive (FP). The precision and recall values for a specific BB, say $b_{i}$, are calculated using the accumulated TP and FP values of all BBs with higher rank than $b_{i}$. The precision-recall curves often show a non monotonous behavior, i.e., they go up and down. This is caused by stochasticity in the ordering of the ranked BBs. To reduce the impact of this effect, the interpolated AP is used to smoothen the shape of the precision-recall curve. Using 10 interpolation points plus zero gives

(4) $$AP=\frac{1}{11}\sum_{r\in \{0,0.1,\ldots ,1\}}p_{interp}\left(r\right)$$

with

(5) $$p_{interp}\left(r\right)=\max_{\hat{r}:\hat{r}\geq r}p\left(\hat{r}\right).$$

$p(\hat{r})$ is the precision at recall $\hat{r}$. Equation (5) results in the desired smoothening of the precision-recall curve.

The mAP is the average value of AP of every detection class. Here, the detection classes were only the class ‘fox’, and consequently, C=1 and Equation (1) resulted in:

mAP=AP(c).

Intersection over Union (IoU) was used to determine the values TP and FP (see Equation (6)). A detection is true positive if IoU≥0.5 and false positive if IoU<0.5. If an image is labeled and the model does not detect anything, it is false negative.

(6) $$IoU=\frac{area\left(BB_{p}\right)\cap area\left(BB_{gt}\right)}{area\left(BB_{p}\right)\cup area\left(BB_{gt}\right)}$$

with IoU: Intersection over Union, $BB_{p}$: predicted bounding box (BB) from the model, $BB_{gt}$: ground-truth bounding box (e.g., manually labeled).

The trained object detection algorithm delivered for each image, whether a fox is on the image, and if so, the confidence of the detection and the position of the bounding box (center x, center y, width, and height), standardized between 0 and 1.

### 2.6. Automatic Evaluation: Converting Bounding Box Values to Movement Patterns

We used the following methods to infer movement patterns from bounding box data:

1. center y versus center x plot over a time period;
2. vector norm over time.

The center *y* versus center *x* plot in the considered time period reveals places of residence in the cage and was used to create a heat map with the likelihood of residence.

The movement vector of the fox was determined using the movement of the center of the bounding box between two consecutive frames, with the coordinates $\left(x_{f},y_{f}\right)$ and $\left(x_{f+1},y_{f+1}\right)$. Hence, the distance covered by the fox between two frames corresponds to the vector norm.

(7) $$m_{f,f+1}=\sqrt{{\left(x_{f+1}-x_{f}\right)}^{2}+{\left(y_{f+1}-y_{f}\right)}^{2}}$$

The mean vector norm over a time period shows the movement activity of a fox in this period.

(8) $${\bar{m}}_{t}=\frac{1}{F}\sum_{f=1}^{F-1}m_{f,f+1}$$

with mean vector norm ${\bar{m}}_{t}$, time period *t*, and number of frames *F* in *t*. The maximum of the mean vector norm for different time periods and different movement behaviors can be used to determine thresholds for different activity levels. We considered three activity levels:

- high active: bounding box moves; change of location of the fox, e.g., walking, running
- active: bounding box moves little; no change of location of the fox, such as rotation inside the bounding box or minimal movements, e.g., scratching on the ground, stretching
- inactive: bounding box not moving; no movement of the fox, e.g., lying, sitting, or standing still

### 2.7. Automatic Evaluation of Video Data

We evaluated the videos using the trained fox detector as described above. The following steps were implemented for video analysis:

1. frame extraction (5 frames per second);
2. fox detection on each frame;
3. convert detection data into movement data.

**Figure 2:**
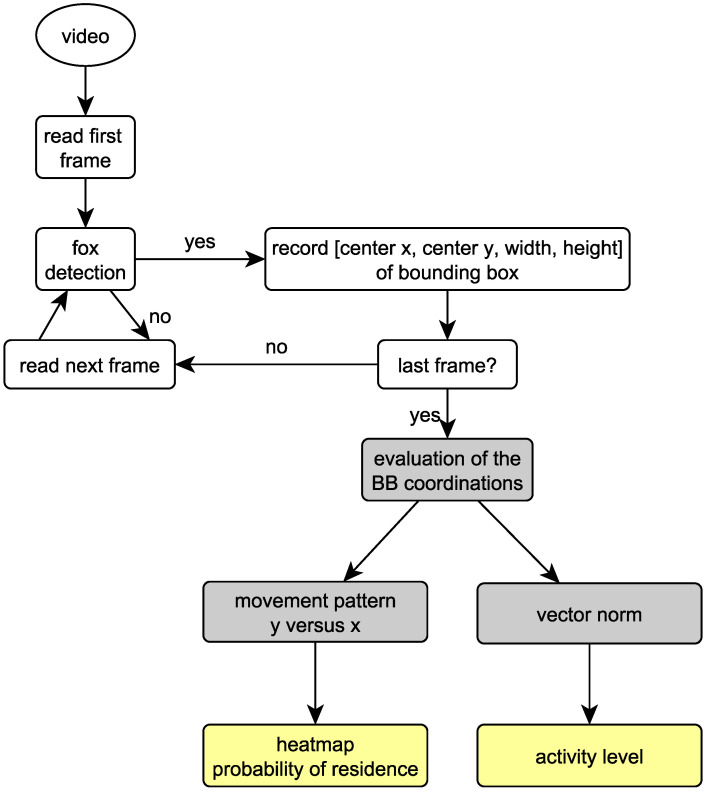
Fox detection and generation of movement patterns. White: evaluation of video data. Grey: evaluation of bounding box (BB) coordination. Yellow: results.

The evaluation process is depicted in Figure 2.

**Figure 3:**
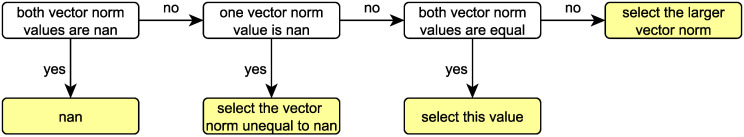
Decision tree to choose the common vector norm for the common evaluation of the two cameras, with nan: not a number. White: properties of both vector norm values. Yellow: selected value.

For the joint evaluation of both cameras of the same animal, each video was evaluated separately for the same time. The two vector norm values were compared for the common evaluation and selected according to the decision tree shown in Figure 3. Note that, in the case of a mismatch between the cameras, the larger vector norm is chosen.

## 3. Results

### 3.1. Model Training and Evaluation

**Figure 4:**
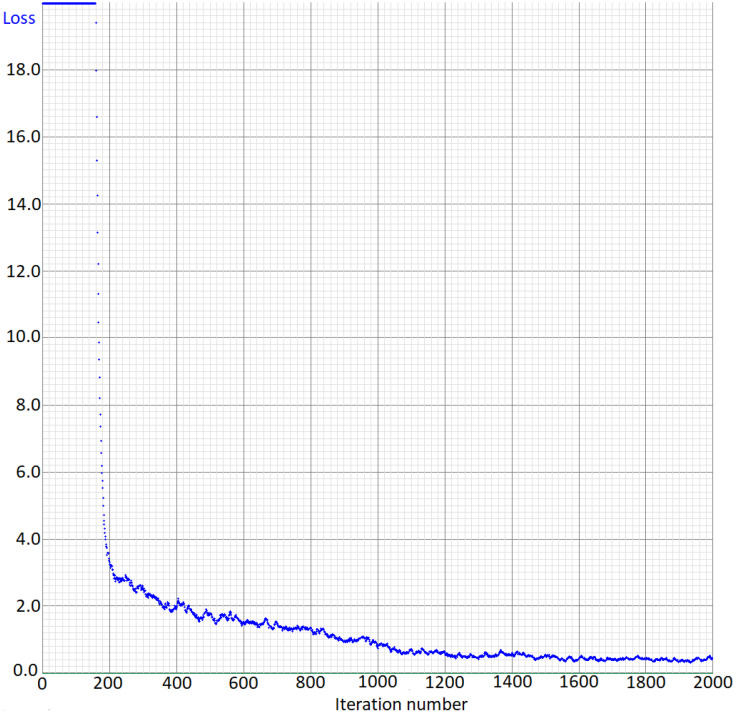
Loss curve of the training of the fox detection model.

The loss curve shows the error on the training set during training (Figure 4). As seen in the Figure, the error decreases sharply during the first 1000 training iterations. Then, the error fluctuates around 0.45.

To verify the trained model, the algorithm was evaluated on the test set with 1473 images, and the results are shown in Table 2. The recall is 99.93%, the precision and mAP are 100%, respectively, the averageIoU is 91.4, and the detection speed is 73.31 ms per image. This demonstrates that the trained model achieves high precision. Moreover, the detection speed is sufficient for a real-time detection with 5 fps, which was used in this work for the generation of movement pattern.

**Figure 5:**
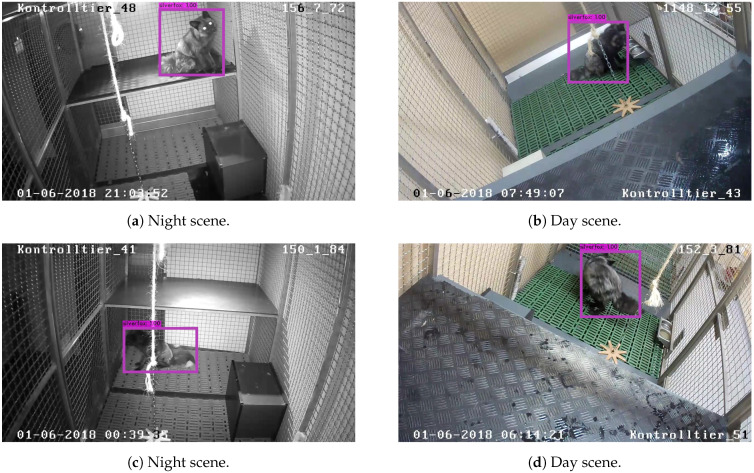
Detection of foxes in single frames.

Figure 5 shows four examples of fox detection. The detection works for night and day images, as shown in Figure 5a, Figure 5c and Figure 5b, Figure 5d, respectively.

### 3.2. Motion Monitoring

To detect motion patterns, we have analyzed video data of fox observations. In the following, the evaluation is shown with two videos. Both videos have a run time of half an hour.

Video 1 shows the following behavioral pattern: Until second 1394, the fox is in a very active phase. Most of the time it is permanently walking, even partly running through the cage. Only for a short period (between seconds 600 and 800) the behavioral pattern changes; the animal scratches at the floor and moves only slightly. From second 1395 onward, the fox is in the blind spot of the camera.

The behavioral pattern in video 2 is different: Here, the fox sleeps most of the time during the observation period. Until second 400, the fox is active, walking through the cage, this phase of activity is interrupted by short phases of sitting, followed by phase of relative inactivity which lasts for 320 s. During this phase the animal is lying and awake and finally changes into sleeping phase for the rest of the observation period with fox lying on its side.

#### 3.2.1. Residence Patterns

**Figure 6:**
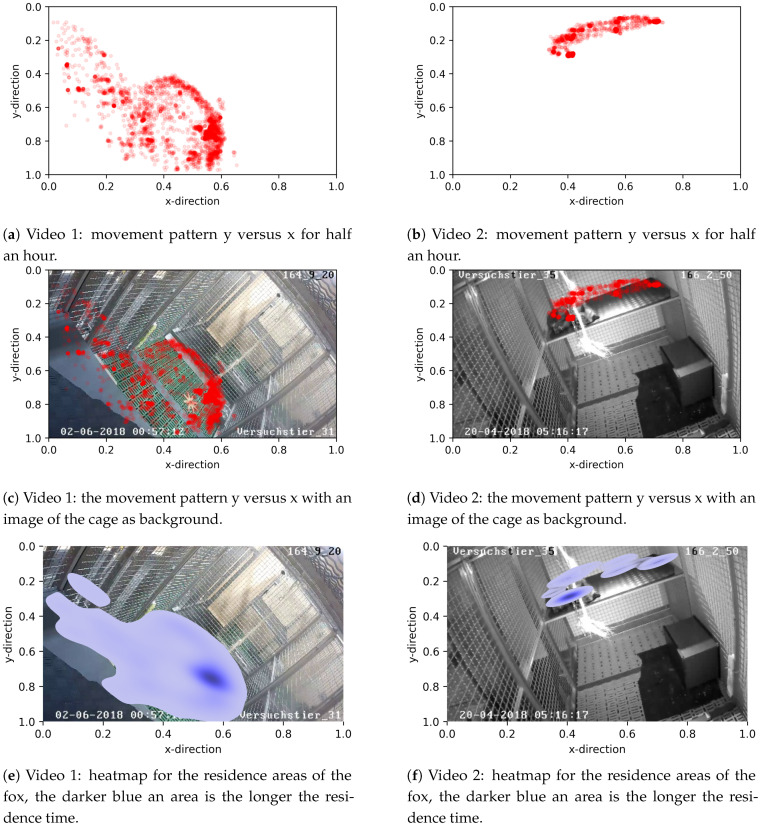
Movement patterns y versus x of video 1 and 2, respectively, with half an hour time period.

One way to study movement patterns of foxes during a given time period is the analysis of places of residence. To this end, the places of residence of the two foxes from videos 1 and 2 for the entire observation period were plotted at a two dimensional scale (y versus x) without (Figure 6a,b) and with an image of the cage as background (Figure 6c,d). It can be seen that the fox in video 1 uses the majority of space in the cage, in contrast to the fox in video 2. The latter remained solely on the platform for the entire observation period.

Subsequently, the x- and y-coordinates were used to generate heat maps for the residence places of foxes (Figure 6e,f), whereby the intensity of the color depicts areas with the higher residence periods. As seen in plot Figure 6e, the dark blue area at position (0.55, 0.8) corresponds to the scratching-on-ground phase in video 1, during this phase the foxes stayed at the same place for a long time. The dark blue area at position (0.4, 0.3) of Plot Figure 6f corresponds to the sleeping place of the fox in video 2.

#### 3.2.2. Activity Detection

**Figure 7:**
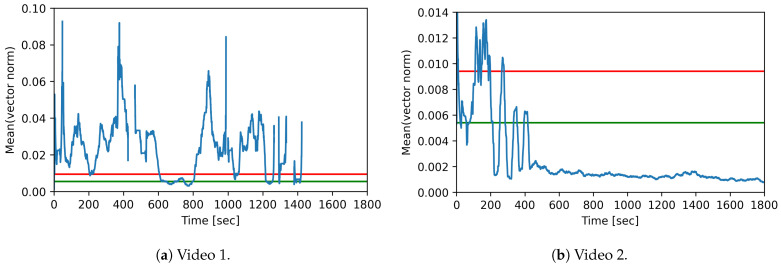
Movement pattern for videos 1 and 2: mean vector norm for 30 sliding window over time, with the thresholds for activity level differentiation. Green: inactive ↔ active. Red: active ↔ high active.

The detection of ‘high active’, ‘active’, and ‘inactive’ phases of the foxes was achieved by using the vector norm of the bounding box center between two sequential video frames. The mean vector norm was calculated using a sliding window with a length of 30 s and a step size of 1 s. Figure 7 illustrates the mean vector norm (in blue) for both videos.

The movement of the fox in video 1 is shown in Figure 7a. The fox moves for a long time and even leaves the camera area for short periods, until it leaves the camera area completely at second 1395. The time span between seconds 600 and 800 shows that the fox moves only slightly. This range corresponds to the scratching-on-ground phase in video 1.

Figure 7b shows the movement of the fox in video 2. The plot shows that the fox moves until second 400 and then stops. This corresponds to the lying and sleeping phase in the video from second 400 onward.

**Figure 8:**
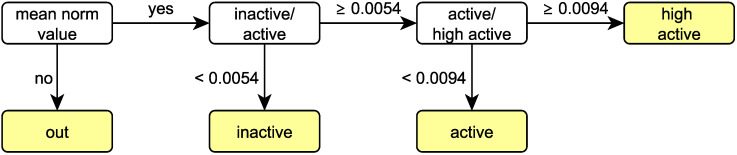
Decision tree for the activity levels using thresholds for the mean norm value.

In order to distinguish between the activity levels, characteristic (known) activities were considered and the maximum of the mean value of the vector norm for a time window of 30 s was determined. For this purpose, different video sequences were extracted for each activity level. For classification we chose thresholds, each between the smallest value of the respective higher activity level and the largest value of the respective lower activity level. These thresholds are displayed in a decision tree (Figure 8).

Figure 7 additionally shows the thresholds for high active ↔ active in red and for active ↔ inactive in green.

As shown in Figure 7a, the mean vector norm is below the threshold active ↔ inactive for 102 s. The duration of the activity level ‘active’ is 205 s and of the activity level ‘high active’ 985 s. In addition, the fox is in the blind spot for 509 s. This corresponds to the activity behavior of the fox in video 1. The phase between seconds 600 and 800, during which the fox scratches the ground and stands partially still corresponds to the activity levels ‘active’ and ‘inactive’, as can be clearly seen in the Figure 7a.

Figure 7b shows that the fox moves on all three activity levels until second 450. Between seconds 200 and 400, three phases can be recognized in which the value is below the threshold active ↔ inactive, these correspond to short phases in which the fox has been sitting. From second 450 onward, the mean vector norm value is permanently below the threshold active ↔ inactive. Altogether, the plot reflects the activity behavior of the fox in video 2.

In this way the duration of the different activity levels can be determined, the values are shown in Table 3.

**Figure 9:**
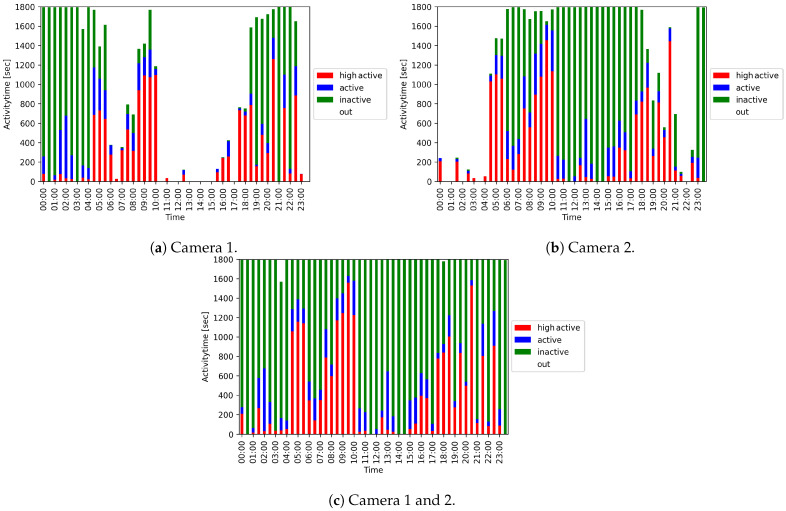
24-h activity overviews of a fox depicted in 30 min time intervals.

We used the duration of the different activity levels to create a 24-h activity overview. Figure 9 shows a 24-h activity overview of a fox in steps of 30 min for the two cameras alone (Figure 9a,b) and for the joint evaluation (Figure 9c). The periods marked with ‘out’ stand for the time, during which the fox was not detected. These are, for example, phases in which the fox is in its hut or in the blind spot. The evaluation of the individual cameras alone leads to gaps, while an almost complete overview can be generated by the joint evaluation of the two cameras.

**Figure 10:**
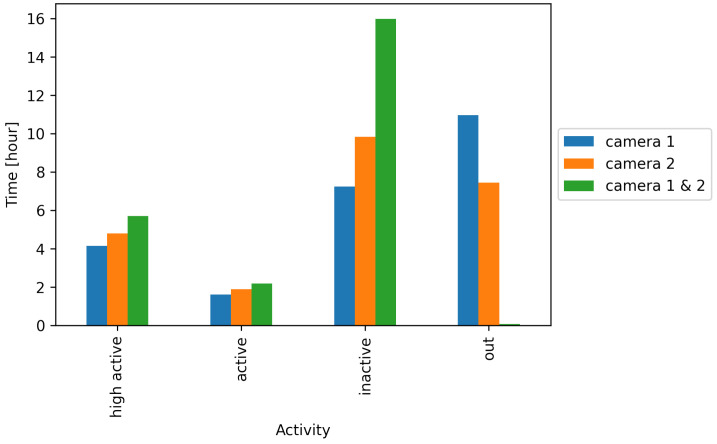
Condensed 24-h activity overview per camera and both cameras evaluated together.

Figure 10 shows the duration of the different activity levels and ‘out’ for the two cameras alone and the joint evaluation. It becomes evident that the duration of ‘out’ can be significantly reduced by the combined evaluation of both cameras.

### 3.3. Comparison of Different Activity Evaluation Methods

Computer evaluation: Five images per second were evaluated. From this, the mean vector norm was calculated once per second. This resulted in 3600 measurements per hour.

The videos were also partially evaluated manually and activity has been captured in the experiments by infra-red motion detectors.

Motion detector data: The motion detectors recorded in 10 s steps, if motion was detected in the last 10 s. This resulted in 360 measurements per hour.

**Figure 11:**
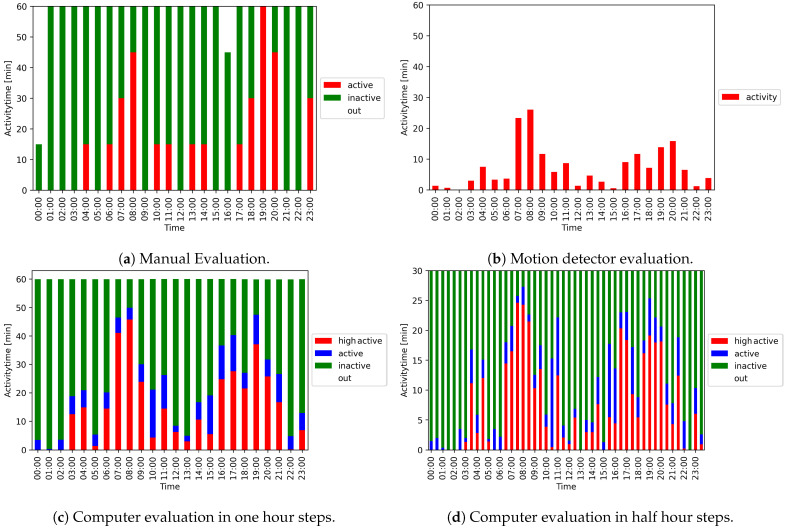
24-h activity overview of a fox using different evaluation methods.

Manually evaluated data: In the manual evaluation, a short video time slice was viewed every 15 min and the respective behavior was extrapolated to the entire 15 min interval, which resulted in four measurements per hour. Figure 11 shows all three evaluation methods for a 24-h activity overview of a fox.

## 4. Discussion

Computer evaluation has proven itself to be useful for fox detection and activity level determination. In our setting, the results with YOLOv4 are very accurate and clearly sufficient for the downstream movement analysis. Therefore, no other object detection software needs to be considered. Furthermore, the animals do not need to be equipped with a sensor or collar, as it was the case in other studies (e.g., [14,20]).

The creation of a residence pattern of the foxes could be used, for instance, to detect preferred residences like the platform or the roof of the hut. This information could be used in new studies to equip the experimental setup with these preferred residences, to improve animal welfare by establishing proper indicators.

Changes in movement patterns can provide insights about animal welfare and health [20,32]. We show that computer vision systems are useful to generate movement patterns.

Our results demonstrate that a computer evaluation of the video data can achieve high precision and can be performed in real-time. A problem is the detection of rarely occurring fox positions that are not included in the training set. To overcome this, the training set may be expanded with rare fox positions.

Another problem was that foxes manipulated the cameras, which were located inside the cages, with the effect that the camera angle changed occasionally, which partially increased the blind spot. The cameras were then re-positioned as soon and as possible. The bias resulting from this situation cannot be eliminated from the current data. However, in further video surveillance activities, one should take care that the camera is properly fixed, maybe outside the cages, with a small hole for the lens in the grid of the cages.

The jointly evaluated video data from both cameras were used to determine the activity levels of the foxes. This led to an almost complete monitoring of the foxes resulting in full activity patterns for different foxes in different time periods (e.g., daily, weekly, day versus night). The duration of motion activity of healthy, normally behaving animals can be used to determine threshold values for normal activity patterns. Consequently, an activity behavior outside these thresholds indicates unfavorable conditions and may, therefore, be helpful to monitor animal welfare indicators.

**Figure 12:**
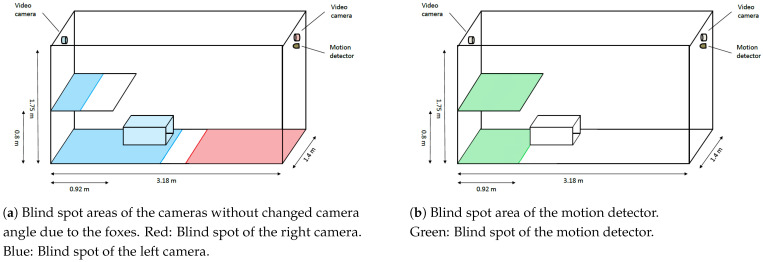
Estimated blind spot areas of the cameras and the infra-red motion detector.

In our setting, the results were comparable to those of manual video assessment and automated motion detection (Figure 11). One challenge for all activity measurement methods is that the fox can be located in a blind spot (Figure 12). This applies for the motion detector in particular, because it covers, by default, only a defined area of the cage. Additionally, each of the two cameras installed only covered a certain area of the cage, but by combining both recordings, the blind spot can be significantly reduced (see Figure 11a,c). Although the trend of activity was generally captured by all methods, computer evaluation had the highest precision. The main reason for this higher accuracy is the number of observations per hour, which is magnitudes higher than for the other approaches (computer evaluation: 3600; motion detector: 360; manual evaluation: 4) and the significantly smaller blind spot area of the cameras compared to the motion detector. The motion detector correctly identified whether the fox is active, but not the complete duration of activity is captured, and it is limited in detecting inactivity or non-detection.

In contrast, manual evaluation allows a precise analysis of activity and non-activity. However, it is restricted due to the short time frame that is used to infer the activity pattern for 15 min, and thus leads to over or under-representation. One example is shown in Figure 11a,c at hours 19–20. Although the activity duration of the fox is 60 min according to the manual evaluation, it is only 47 min after computer evaluation. A comparison of the different methods is provided in Table 4.

Another advantage of both motion detectors and computer evaluation is the possibility to modify the time windows for downstream analyses. These can be seen in Figure 11c,d, where the 24-h overview is shown in hourly and half-hourly steps.

The motion detector detects the activity level ‘active’, whereas manual evaluation can distinguish between ‘active’ and ‘inactive’, and if the fox is in the blind spot.

Finally, most of the different activity levels can be determined using computer evaluation. This method can even distinguish between ‘active’, ‘inactive’, and ‘high active’—and not detectable.

In summary, computer evaluation is clearly the best option to evaluate the videos completely, without the time, labor, and costs required for manual evaluation.

## 5. Conclusions

This study shows an application of YOLOv4 for the automatic detection of foxes and the creation of different movement patterns that can be used for animal behavioral analysis and, thus, animal welfare monitoring.

Compared to other approaches, successfully established computer evaluation offers the huge advantage of seamless data analyses from videos in real-time, without additional cost or personnel effort. Besides the creation of activity level overviews, the detailed data from computer evaluation allow for even more sophisticated analyses, e.g., movement and residence patterns can be derived.

### Outlook and Future Research Possibilities

In this paper, we studied the movement pattern of two foxes exemplary for a certain time span. In order to establish reference data on the behavior of foxes under captive conditions, larger datasets would need to be analyzed.

In this paper, we have distinguished between the different activity levels using a manual definition of thresholds. These thresholds could also be estimated automatically using either unsupervised learning techniques (e.g., clustering) or supervised learning (such as a decision tree based classifier). In a similar fashion, this approach could be extended to detect anomalies in behavior, e.g., in case of disease. Once patterns of normal behavior are established, thresholds could be set and detected, so that a change in behavior, indicative of animal welfare issues, e.g., disease, is automatically detected. Additionally, the interaction with specific objects, such as water and food bowls, or toys—and whether this has any influence on activity patterns—could be included in the computer analysis. To this end, the detector would have to be trained with these objects.

To improve behavioral analysis, the detector can be trained on different body postures of the fox to classify these in addition to its position in the image.

Finally, the approach we demonstrated here could be transferred to other animal species under various settings, e.g., livestock animals in larger barns or even the automated detection of different animal species in wildlife monitoring cameras. Beyond the laboratory environment considered here, the detector also works with images of silver foxes in natural environments. In addition, the detector can easily detect multiple animals at the same time. This feature could be used to study the social behavior and group dynamics of animals. Depending on the considered species, it might even be possible to train the detector to identify and track individual animals using unique features of their appearance.

## Figures and Tables

**Table 1:** Parameters of YOLOv4 fox detection model.

Parameter	Value
input size	416×416
classes	1
maxbatches	2000
filters	18
steps	1600, 1800
learning rate	0.001
batch size	64

**Table 2:** Fox detection test results for the trained model.

	*Recall*	*Precision*	*Average IoU*	*mAP*	*Detection Speed*
	[%]	[%]	[%]	[%]	[ms]
test results	99.93	100	91.4	100	73.31

**Table 3:** Duration of activity levels in seconds for video 1 and video 2.

	High Active	Active	Inactive	Out
video 1	985	205	102	509
video 2	100	191	1509	0

**Table 4:** Potential benefits and disadvantageous of the three different evaluation methods.

Method	+	−
computer	complete (24/7) analysis of animal activity perceptively, behavioral detection based on learning from manual data sets fast and not time-intensive for human resources once established can be further expanded and algorithms may be used for further studies independent of human-bias small amount of data for saving the x- and y-coordinates of the bounding box	only if evaluation is not in real time, or the data has to be saved for further evaluation: large amounts of video storing learning of pattern detection based on manual assignment extensive IT resources required
motion detector	data collection live to the experiment produces relatively small amount of data that needs to be stored independent of human-bias	can only collect yes/no responses to activity, no further analysis possible affected by human-presence (e.g., animal caretakers) as any motion is recorded
manual	detection of activity/inactivity diverse analysis of behavior based on an ethogram	slow and time-intensive for human resources (sample intervals) huge amounts of video storing potential human-bias

## Data Availability

The datasets found or analyzed during the current study are available from the corresponding author on reasonable request.

## Abbreviations

The following abbreviations are used in this manuscript:

AP	average precision
BB	bounding box
CNN	convolutional neural networks
FLI	Friedrich-Loeffler-Institut
FP	false positive
IoU	intersection over union
IT	information technology
mAP	mean average precision
nan	not a number
TP	true positive
YOLO	you only look once
